# Integrative Genome Comparison of Primary and Metastatic Melanomas

**DOI:** 10.1371/journal.pone.0010770

**Published:** 2010-05-24

**Authors:** Omar Kabbarah, Cristina Nogueira, Bin Feng, Rosalynn M. Nazarian, Marcus Bosenberg, Min Wu, Kenneth L. Scott, Lawrence N. Kwong, Yonghong Xiao, Carlos Cordon-Cardo, Scott R. Granter, Sridhar Ramaswamy, Todd Golub, Lyn M. Duncan, Stephan N. Wagner, Cameron Brennan, Lynda Chin

**Affiliations:** 1 Department of Medical Oncology, Dana-Farber Cancer Institute and Harvard Medical School, Boston, Massachusetts, United States of America; 2 Institute of Molecular Pathology and Immunology of the University of Porto (IPATIMUP), University of Porto, Porto, Portugal; 3 Belfer Institute for Applied Cancer Science, Dana-Farber Cancer Institute, Boston, Massachusetts, United States of America; 4 Dermatopathology Unit, Department of Pathology, Massachusetts General Hospital, Harvard Medical School, Boston, Massachusetts, United States of America; 5 Department of Dermatology, Yale University School of Medicine, New Haven, Connecticut, United States of America; 6 Department of Pathology, Columbia University, New York, New York, United States of America; 7 Department of Pathology, Brigham and Women's Hospital, Boston, Massachusetts, United States of America; 8 Massachusetts General Hospital Cancer Center, Boston, Massachusetts, United States of America; 9 The Broad Institute of MIT and Harvard and Dana-Farber Cancer Institute, Boston, Massachusetts, United States of America; 10 DIAID, Department of Dermatology, Medical University of Vienna and Center of Molecular Medicine, Austrian Academy of Sciences, Vienna, Austria; 11 HOPP, Department of Neurosurgery, Memorial Sloan-Kettering Cancer Center, New York, New York, United States of America; 12 Department of Dermatology, Brigham and Women's Hospital and Harvard Medical School, Boston, Massachusetts, United States of America; Roswell Park Cancer Institute, United States of America

## Abstract

A cardinal feature of malignant melanoma is its metastatic propensity. An incomplete view of the genetic events driving metastatic progression has been a major barrier to rational development of effective therapeutics and prognostic diagnostics for melanoma patients. In this study, we conducted global genomic characterization of primary and metastatic melanomas to examine the genomic landscape associated with metastatic progression. In addition to uncovering three genomic subclasses of metastastic melanomas, we delineated 39 focal and recurrent regions of amplification and deletions, many of which encompassed resident genes that have not been implicated in cancer or metastasis. To identify progression-associated metastasis gene candidates, we applied a statistical approach, Integrative Genome Comparison (IGC), to define 32 genomic regions of interest that were significantly altered in metastatic relative to primary melanomas, encompassing 30 resident genes with statistically significant expression deregulation. Functional assays on a subset of these candidates, including *MET*, *ASPM*, *AKAP9*, *IMP3*, *PRKCA*, *RPA3*, and *SCAP2*, validated their pro-invasion activities in human melanoma cells. Validity of the IGC approach was further reinforced by tissue microarray analysis of Survivin showing significant increased protein expression in thick versus thin primary cutaneous melanomas, and a progression correlation with lymph node metastases. Together, these functional validation results and correlative analysis of human tissues support the thesis that integrated genomic and pathological analyses of staged melanomas provide a productive entry point for discovery of melanoma metastases genes.

## Introduction

Cutaneous melanoma arises primarily from neural crest-derived epidermal melanocytes [1]. A reflection of melanoma's intense metastatic propensity is the fact that the metastatic risk is measured on the scale of millimeters, where a tumor thickness of only 4 mm predicts a high risk of cancer dissemination and death [1]. When localized to the skin, cutaneous melanoma is largely curable by surgical excision, whereas metastatic melanoma carries a median survival of 6–9 months [1]. The recent success of targeted therapies in melanoma [2] substantiates the view that a more comprehensive examination of the genetic events governing melanoma development, particularly its metastatic potential, may lead to more effective therapies directed against this disease.

The molecular basis of melanoma genesis and progression has not been fully elucidated. Several validated genetic mutations (i.e., documented DNA structural alterations) responsible for melanocytic transformation have been described, including deletion of the 9p21 CDKN2A familial melanoma locus encoding the tumor suppressors INK4A and ARF, as well as amplification of *MITF* as a lineage-specific oncogene [3]. Activation of MAPK signaling is frequently observed in melanocytic neoplasms through activating mutations of *BRAF* or *NRAS* in cutaneous melanoma [3] or mutations of the heterotrimeric guanine nucleotide-binding protein GNAQ in uveal melanoma [4]. An integrative cross-species comparative oncogenomic analysis identified *NEDD9*, a member of the p130CAS family, as a target of a recurrent 6p gain; and functional studies verified its role as a *bona fide* melanoma metastasis gene [5] involved in mesenchymal cell movement [6]. Recently, Nedd9 expression has also shown to be required for breast cancer metastasis *in vivo* [7]. In addition to this handful of genes, genomic characterization of metastatic melanomas and melanoma cell lines have uncovered many regions of recurrent, non-random chromosomal copy number aberrations (CNAs) with few recognizable or validated cancer-relevant genes, pointing to the potential existence of many yet-to-be-discovered genetic events driving melanoma pathogenesis [8], [9].

DNA copy number aberrations would be expected to be retained throughout the life history of a cancer cell. These aberrations are presumed to include drivers and passengers as well as events responsible for the initiation and/or progression of disease. As such, there are significant challenges in the identification of metastasis-relevant alterations. In this study, we examined the genomes of a collection of clinically annotated primary and metastatic melanomas. Not surprisingly, given the well-recognized heterogeneous nature of primary melanoma, many more genomic alterations were definable in metastatic melanomas, providing an opportunity for comparative analyses to identify events that are enriched for during metastatic progression. To this end, using an Integrative Genome Comparison (IGC) approach, we defined a short list of 30 candidates that showed increased expression and resided within regions of amplification in metastatic melanomas. Functional characterization and correlative analysis of human tissues supported a role for these candidates in cell invasion.

## Results

### The melanoma genome is highly rearranged and heterogeneous

**Figure 1:**
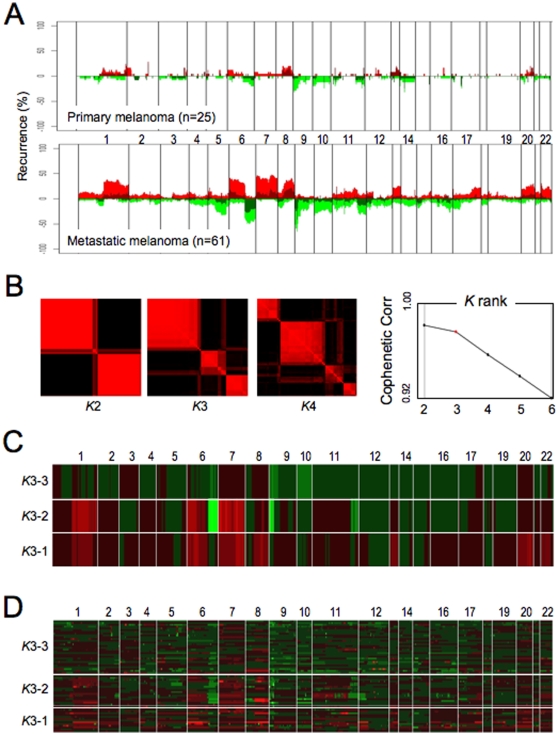
Array-CGH characterization of the primary and metastatic melanoma genomes. (**A**) Summary of genomic profiles of primary and metastatic melanomas and the recurrence of chromosomal alterations. Recurrence of CNAs across the samples in segmented data (y axis) is plotted for each probe evenly aligned along the x axis in chromosomal order. The percentage of tumors harboring gains, amplifications, losses and deletions for each locus is depicted according to the following scheme: dark red (gains with a log_2_ ratio > = 0.15) and green (loss with a log_2_ ratio < = −0.15) and are plotted along with bright red (amplifications with a log_2_ ratio ≥ 0.4) and bright green (deletions with log_2_ ratio ≤−0.4). (**B**) Consensus matrices show how often samples are assigned to the same clusters during 100 repetitions of gNMF, computed at K = 2–4 for the 61 metastatic melanoma dataset. Each pixel represents how often a particular pair of samples clusters together, colored from 0% (black, samples are never in the same cluster) to 100% (red, samples are always in the same cluster). Ranks 2 and 3 classification show stable assignments into 2 and 3 blocks, respectively; in contrast, rank 4 assignments are disrupted. Cophenetic correlation coefficients for hierarchically clustered matrices in B. Valid clustering should show correlation close to 1. (**C**) gNMF classification with rank K = 3 identifies three distinct subgroups. Array-CGH profiles of 61 metastatic melanomas were subjected to gNMF analyses (100 repetitions). Y axis indicates the centroid of three subgroups identified by gNMF. X axis coordinates represent genomic map order (from chromosome 1 to chromosome 22). The colors denote gained (red) or deleted (green) chromosome material. (**D**) Heat-map plot showing discrete CNAs within all samples, with the X axis coordinates represent genomic map position and Y axis indicates individual samples of the three subgroups identified by gNMF. Red represents chromosomal gain or amplification, and green denotes chromosomal loss or deletion.

Using an established oligo-microarray platform offering a median resolution of 50 kb [10], we compiled array-CGH profiles on 25 primary cutaneous and 61 metastatic melanoma specimens. The clinical and histopathologic characteristics of these samples are summarized in Supplemental Tables S1 and S2, and the array-CGH profiles are available online at GEO under super-series accession #GSE7606. Raw array-CGH profiles were processed by a modified circular binary segmentation (CBS) algorithm [11], [12], and copy number aberrations (CNAs), represented by genomic segments bounded by statistically significant copy number transition points, were defined in each profile (see Methods). When viewed in skyline recurrence plots (Figure 1A), the overall patterns of CNAs in metastatic profiles agreed well with major and frequent events previously reported in melanoma [8], [13], [14], [15], including gains on 1q, 6p, 7, 8q, 17q, 20, and 22q, as well as losses on 6q, 8p, 9, 10, and 11q. In contrast, primary melanomas harbored far fewer genomic alterations detectable by array-CGH. Indeed, by measuring the breakpoints of the genome with altered copy number events (see Supplemental Figure S1 legend), one could demonstrate such statistically significant increase in discernable genomic events from primary to metastatic melanomas (Supplemental Figure S1; p = 5×10^−5^).

In view of the highly rearranged nature of the metastatic melanoma genome, we next asked whether metastases were comprised of distinct genomic subclasses by genomic non-negative matrix factorization (gNMF), an unsupervised classification algorithm modified for array-CGH data [16], [17]. Notably, strong Cophenetic correlations were observed when gNMF classified these profiles into 2 or 3 subclasses (e.g. Rank *K*2 and *K*3 classification, respectively); whereas Rank *K*4 showed a sharp drop in correlation (Figure 1B). Thus, gNMF classification defined three stable molecular subclasses among the metastatic samples. Examination of key features of these subclasses revealed that the *K*3-1 profile was characterized by gains of chromosomes 1q, 6p, 7, 8q, 13, 20 and 22p, whereas *K*3-2 showed prominent 1q, 6p, 7 and 8q gains accompanied by loss of 6q, 9p and 11q and *K*3-3 presented with a general hypoploidy profile (Figure 1C). These patterns were consistent with the expression heatmap of the samples grouped according to their subclass assignment (Figure 1D). As melanoma metastases have reportedly been classified into two distinct transcriptional subtypes, and those subgroups were significantly correlated with clinically-relevant endpoints, including patient survival [18], we asked whether this DNA-based classification was associated with any clinical parameters. Notably, the subclass assignments did not correlate with metastatic site, age or gender (data not shown; Supplemental Table S1). Instead, when intersected with survival outcome available on a subset of these samples, *K*3-3 subclass appeared to have a significant survival advantage by Kaplan-Meier analysis (Supplemental Figure S2), suggesting that these genomic subclasses likely represent biologically- and clinically-relevant subpopulations.

### Defining recurrent regions of amplification and deletion

Further analysis of the focal alterations in the highly rearranged genomes of primary melanomas and metastases delimited the boundaries of informative Minimal Common Regions (MCRs) using a set of heuristically defined rules, including recurrence in 2 or more samples of a CNA spanning regions less than 2Mb in size with a peak log_2_ ratio greater than 1.0 (see Methods). In the primary melanomas, this analysis defined 13 MCRs comprising 6 amplifications with a median size of 1.03 Mb (range 0.075–1.97 Mb) containing a total of 84 known genes, and 7 deletions with a median size of 0.32 Mb (range 0.098–0.94 Mb) containing 39 genes (Table 1). In comparison, analysis of the metastasis profiles defined 39 MCRs comprising 24 amplifications with a median size of 0.78 Mb (range 0.046–1.59 Mb) containing a total of 276 known genes, and 15 deletions with a median size of 0.53 Mb (range 0.035–1.7 Mb) encompassing 78 genes (Table 1). Although the cytological bands of MCRs in primary and metastatic melanoma do not entirely overlap, these do not necessarily represent unique events to one or the other melanoma type since they can be present as regions of larger amplifications or deletions or lower amplitude changes (and thus can be excluded from the list of informative MCRs due to the strict criteria used to define these events). The identification of regions of genomic alteration enriched in primary or metastatic melanoma is discussed below.

**Table 1:** High-confidence MCRs in melanoma primary and metastastic samples.

Primary Melanomas									
MCR#	Cytobands	Start	End	Width (bp)	Peak	# Tumors	# Genes	*Candidates*	known CNV*?
1	1q21.1	142,480,203	144,454,599	1,974,396	1.14	2	40	*PDE4DIP, BCL9*	yes
2	1q24.1	162,608,779	163,764,545	1,155,766	1.51	2	13	*GPA33*	partial
3	2q31.1	175,489,973	176,859,506	1,369,533	1.16	6	15	*HOXD11, HOXD13, CHN1*	no
4	5p13.3	31,589,913	32,485,015	895,102	1.15	2	6		yes
5	11q24.2	125,577,665	125,652,604	74,939	1.14	4	4		no
6	20q13.33	59,983,746	60,209,329	225,583	1.03	3	6	*SS18L1*	partial
7	1p21.2	101,168,629	101,448,588	279,959	−1.06	2	3		no
8	6q27	169,921,072	170,019,433	98,361	−1.31	3	5		no
9	9p24.1	5,899,734	6,247,371	347,637	−1.17	2	5		no
10	11q21	93,552,953	93,872,148	319,195	−1.10	2	6	*MRE11A*	no
11	11q23.3	120,465,281	120,683,610	218,329	−1.43	4	3		no
12	14q21.1	37,749,185	38,688,955	939,770	−1.46	6	8		partial
13	15q26.3	98,987,639	99,630,115	642,476	−1.24	4	9		yes

Metastatic Melanomas									
MCR#	Cytobands	Start	End	Width (bp)	Peak	# Tumors	# Genes	*Candidates*	known CNV?
1	1p31.2	67,865,391	68,606,911	741,520	1.11	2	7	*GADD45A*	no
2	1p12–11.2	119,648,460	120,857,214	1,208,754	1.11	4	27	*NOTCH2*	yes
3	1q21.1	142,606,559	143,875,807	1,269,248	2.16	7	30		yes
4	2q32.2	191,190,685	191,660,535	469,850	1.01	2	5	*STAT1*	no
5	4q22.1	89,799,529	90,000,656	201,127	1.34	3	2		no
6	5p15.33	235,454	1,275,534	1,040,080	1.22	7	21	*NKD2*	yes
7	5p13.3	31,589,913	32,485,015	895,102	1.88	6	6		partial
8	5q31.3	140,456,938	140,607,613	150,675	1.21	4	18		yes
9	6p25.3	295,181	1,258,151	962,970	1.26	10	7	*FOXQ1*	yes
10	7p15.2	26,923,158	26,995,432	72,274	1.02	9	8	*HOXA9, HOXA11*	no
11	7q21.3	96,455,437	97,489,267	1,033,830	1.61	9	12	*ASNS, OCM*	partial
12	7q22.1	99,118,939	99,165,774	46,835	1.53	5	1		No
13	8q23.1–23.2	110,324,340	110,608,305	283,965	1.25	13	4		No
14	11q12.1	56,705,905	56,850,264	144,359	1.27	2	4	*AGTRL1, TNKS1BP1*	No
15	11q13.3–13.4	69,334,129	70,823,161	1,489,032	1.01	6	12	*FGF3, CCND1, CTTN*	No
16	12p13.32	4,332,302	4,468,861	136,559	1.06	5	4	*FGF23, FGF6*	No
17	12p12.3	18,125,215	19,420,372	1,295,157	1.06	5	6	*PIK3C2G*	No
18	12q14.1	56,306,778	56,477,913	171,135	4.41	4	12	*CDK4, OS-9, CENTG1, SAS*	No
19	12q15	66,881,510	67,951,061	1,069,551	2.88	2	12	*MDM2, RAP1B*	No
20	13q22.3–31.1	76,477,880	78,071,471	1,593,591	1.20	4	7	*EDNRB*	partial
21	16q13	55,674,611	56,373,793	699,182	1.03	2	20	*GPR56*	No
22	16q22.1	66,420,083	66,520,918	100,835	1.30	5	6		No
23	19p13.2–13.13	11,546,524	12,601,182	1,054,658	1.08	3	33		partial
24	22q13.1–13.2	39,131,326	39,950,686	819,360	1.17	2	12	*MKL1, EP300*	No
25	2p25.2	4,731,218	5,784,905	1,053,687	−1.10	2	6		partial
26	5q15	92,955,495	93,101,775	146,280	−1.30	2	3		No
27	5q15	93,223,093	94,811,785	1,588,692	−1.03	3	9		partial
28	5q21.3–22.1	108,699,117	110,439,871	1,740,754	−1.11	5	7		partial
29	5q33.3–34	159,762,714	160,690,553	927,839	−1.04	7	4		partial
30	6p25.3	295,181	430,239	135,058	−1.43	3	3	*IRF4*	yes
31	9p24.1	4,850,884	5,547,670	696,786	−2.10	14	11	*JAK2*	partial
32	9p21.3	21,471,141	21,998,963	527,822	−2.15	26	6	*CDKN2A, CDKN2B*	partial
33	10q23.32	93,657,523	93,799,360	141,837	−1.23	4	2		No
34	12q21.1–21.2	74,103,510	74,709,669	606,159	−1.03	3	4	*GLIPR1*	No
35	12q23.1	95,176,246	95,849,465	673,219	−1.42	5	5		No
36	14q32.12	91,117,109	91,405,833	288,724	−1.04	2	4	*FBLN5*	No
37	16q24.3	88,589,893	88,650,942	61,049	−1.02	3	6	*GAS8*	yes
38	19q13.42	60,820,660	60,855,625	34,965	−1.23	3	4		No
39	22q13.31	44,560,277	44,767,323	207,046	−1.01	3	4	*WNT7B*	No

*MCRs were mapped to regions of known copy number varation.

Of the genes residing within metastases MCRs boundaries (Table 1), many were linked to networks of relevance to carcinogenesis and metastasis. For example, a significant number of genes were involved in G1/S cell cycle transition and in p53-dependent apoptosis (Table 1; MetaCore™ analysis, p<0.01), including p14^ARF^, p16^INK4A^ and p15^INK4B^, which were deleted as part of the recurrent 9p21 locus deletion, as well as CDK4 and MDM2, both of which were recurrently amplified in metastatic melanoma (Supplemental Figure S3). Additionally, MetaCore™ analysis identified components of networks governing cell adhesion, motility and cell matrix assembly that were significantly represented among genes mapping to the metastases MCRs (p<0.01). For example, *LIPRIN* (*PPFIA1*), a gene known to enhance cell matrix interaction, and *Contractin* (*CTTN*), a gene implicated in squamous cell carcinoma migration and metastasis [19], [20], were both recurrently amplified in metastatic melanoma. Conversely, *Fibulin 5* (*FBLN5*), shown to enhance cell adhesion [21], was recurrently deleted in the metastatic samples (Table 1).

### IGC analysis of primary and metastastic melanoma genomes

**Figure 2:**
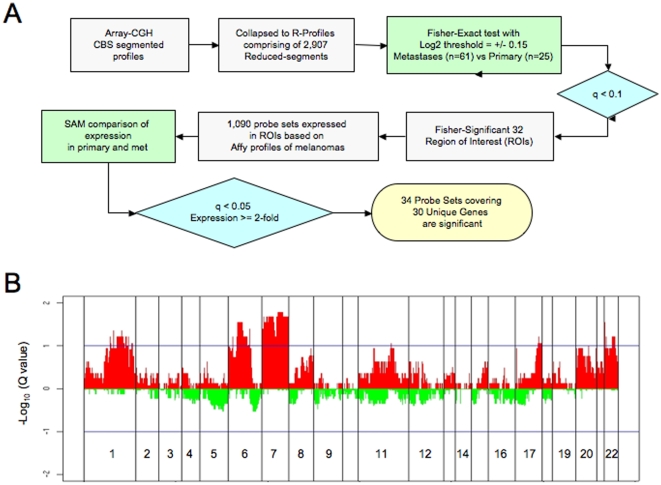
Integrative genomics identify high-confidence metastasis candidate melanoma genes. (**A**) Flow chart of integrating copy number and expression analysis to compare primary and metastastic melanoma genomes. (**B**) Whole genome q-value profiles based on Fisher's Exact Test between primary and metastastic melanomas. X axis coordinates represent genomic map position and Y axis indicates q-value log10 of Fisher's Exact Test between primary and metastastic melanomas at each R-segment.

Evolution from primary to metastatic disease is expected to be accompanied by the acquisition of, or selection for, genomic and genetic events that confer biologic capabilities necessary for mestastasis [22]. We thus hypothesized that CNAs observed in metastasis but not detected in primary disease would be more likely to represent potential drivers of metastasis. To define such events, we adopted an integrative genome comparison approach to define genes that were statistically different between primary and metastatic samples based on DNA and RNA data (Figure 2A). First, we employed a statistical test, Fisher-Exact, to delineate regions that were differentially altered in metastatic versus primary melanoma. Briefly, we collapsed all CBS-processed array-CGH profiles of primary or metastatic cohorts down to 2,907 reduced-segments (hereafter as “R-segments”) to generate two R-segment profiles corresponding to primary and metastatic melanoma genomes, respectively. For each R-segments above noise threshold (i.e. Log2 of +/−0.15), Fisher's Exact test p-values were calculated and corrected for multiple testing (see Methods) to define statistically significant events that were different between these two classes. At a false discovery rate (FDR) of 10% (q< = 0.1), 300 R-segments spanning 32 contiguous regions of interest (ROIs) were found to be preferentially gained in metastatic relative to primary melanomas in a non-random fashion (Figure 2B). Many of these 32 ROIs clustered predominantly in several chromosomal regions, including 1q, 6p, 7, 17q and 22, and many of the regions were gained in poor-prognosis *K*3-1 and *K*3-2 subclasses (Figure 1C and 1D). Of note, no R-segments were found to be preferentially gained in primary relative to metastatic melanomas and no regions of loss were significantly different between primary and metastasis.

Next, we sought to determine whether genes resident in regions of genomic alterations exhibited a pattern of expression reflective of the underlying copy number aberrations. That is, metastasis candidate genes resident in regions that are preferentially gained in metastatic melanomas would be expected to show upregulation on mRNA level when compared to primary melanomas. Accordingly, we utilized the well-established SAM algorithm to identify those genes resident within the 32 Fisher-significant ROIs that exhibited overexpression patterns in metastastic melanomas relative to primary disease. Specifically, of the 1090 Affymetrix probe sets deemed expressed (see Methods) within these 32 ROIs, SAM analyses identified 676 probe sets that showed significant overexpression in metastases (FDR< = 0.05). These 676 probes were furthered ranked by the relative fold change of expression to select the top 34 probes corresponding to 30 unique annotated genes exhibiting at least 2-fold overexpression in metastases (Table 2). A number of these genes mapped to chromosome 7, whose gain has been linked to metastasis and poor prognosis in patients with non-small cell lung cancer and peripheral nerve sheath tumors [23], [24]. Although a number of these candidates in Table 2 mapped to known regions of germline CNV, we did not exclude these from further consideration since well-validated cancer relevant genes have been known to reside within regions of germline CNV [25].

**Table 2:** The integration of copy number and expression analysis to compare primary and metastastic melanoma genomes identifies 30 unique genes amplified and overexpressed in metastastic melanoma compared to primary melanoma.

Chr	R-Segments			Primary vs Metastasis by SAM			Gene			known CNV*?
	Start (bp)	End (bp)	Width (bp)	Probes	Rel Exp	q value	Symbol	Gene ID	Description	
1	189,881,478	193,480,076	3,598,598	219918_s_at	2.63	0.00	ASPM	259266	asp-like, microcephaly associated (Drosophila)	yes
6	29,678,435	30,145,591	467,156	216229_x_at	2.02	0.00	HCG2P7	80867	HLA complex group 2 pseudogene 7	yes
6	31,649,132	31,733,853	84,721	212384_at	2.08	0.00	BAT1	7919	HLA-B associated transcript 1	yes
7	6,503,371	10,915,738	4,412,367	209507_at	2.37	0.00	RPA3	6119	replication protein A3, 14kDa	No
7	16,413,351	17,606,374	1,193,023	217979_at	2.75	0.00	TM4SF13	27075	transmembrane 4 superfamily member 13	No
7	21,258,989	24,511,806	3,252,817	203820_s_at	2.60	0.00	IMP-3	10643	IGF-II mRNA-binding protein 3	No
7	26,009,673	26,923,158	913,485	204362_at	2.35	0.00	SCAP2	8935	src family associated phosphoprotein 2	No
7	26,009,673	26,923,158	913,485	201091_s_at	2.21	0.00	CBX3	11335	chromobox homolog 3	No
7	31,602,638	38,147,471	6,544,833	204051_s_at	3.03	0.00	SFRP4	6424	secreted frizzled-related protein 4	No
7	31,602,638	38,147,471	6,544,833	212792_at	2.31	0.00	KIAA0877	23333	KIAA0877 protein	No
7	31,602,638	38,147,471	6,544,833	202904_s_at	2.07	0.00	LSM5	23658	LSM5 homolog, U6 small nuclear RNA associated	No
7	55,852,994	55,943,507	90,513	205194_at	2.36	0.00	PSPH	5723	phosphoserine phosphatase	No
7	64,310,010	72,299,706	7,989,696	213460_x_at	2.18	0.00	WBSCR20C	55695	Williams Beuren syndrome chromosome region 20C	yes
7	73,393,701	76,470,379	3,076,678	213670_x_at	2.25	0.00	WBSCR20B	155400	Williams-Beuren Syndrome critical region protein 20, copy B	yes
7	89,659,035	96,294,973	6,635,938	204873_at	2.03	0.00	PEX1	5189	peroxisome biogenesis factor 1	No
7	89,659,035	96,294,973	6,635,938	209278_s_at	5.37	0.00	TFPI2	7980	tissue factor pathway inhibitor 2	No
7	89,659,035	96,294,973	6,635,938	204688_at	2.01	0.00	SGCE	8910	sarcoglycan, epsilon	yes
7	89,659,035	96,294,973	6,635,938	215483_at	2.26	0.00	AKAP9	10142	A kinase (PRKA) anchor protein (yotiao) 9	yes
7	89,659,035	96,294,973	6,635,938	212094_at	2.44	0.00	PEG10	23089	paternally expressed 10	No
7	96,294,973	97,126,111	831,138	205047_s_at	2.51	0.00	ASNS	440	asparagine synthetase	yes
7	97,586,138	99,133,331	1,547,193	213479_at	3.27	0.00	NPTX2	4885	neuronal pentraxin II	No
7	99,463,598	99,609,889	146,291	220954_s_at	2.37	0.00	PILRB	29990	paired immunoglobin-like type 2 receptor beta	No
7	100,130,869	101,618,306	1,487,437	205586_x_at	2.03	0.01	VGF	7425	VGF nerve growth factor inducible	No
7	105,325,416	106,944,473	1,619,057	206529_x_at	2.25	0.00	SLC26A4	5172	solute carrier family 26, member 4	No
7	106,993,757	107,808,465	814,708	202843_at	2.19	0.00	DNAJB9	4189	DnaJ (Hsp40) homolog, subfamily B, member 9	No
7	109,897,338	112,000,722	2,103,384	202147_s_at	2.05	0.00	IFRD1	3475	interferon-related developmental regulator 1	No
7	115,794,692	116,151,838	357,146	203510_at	3.15	0.00	MET	4233	met proto-oncogene	No
7	127,965,509	128,048,784	83,275	214845_s_at	2.04	0.00	CALU	813	calumenin	No
17	61,638,666	62,311,370	672,704	213093_at	2.12	0.00	PRKCA	5578	protein kinase C, alpha	No
17	73,732,314	75,521,030	1,788,716	202095_s_at	2.20	0.00	BIRC5	332	baculoviral IAP repeat-containing 5 (survivin)	No

*MCRs were mapped to regions of known copy number varation.

### Metastasis candidates promote invasion *in vitro*

Among the 30 candidate metastasis genes is *MET*, a receptor tyrosine kinase (RTK) whose overexpression has been correlated with progression in multiple cancer types, including melanoma [26]. Indeed, in a *Met*-driven transgenic mouse model comprised of tyrosinase-driven *rtTA* and tet-*Met* transgenes on the *Ink4a/Arf* null background (*Tyr-rtTA;Tet-Met;Ink4a/Arf^−/−^*, hereafter “iMet”), activation of Met signaling in melanocytes engendered a metastatic melanoma phenotype *in vivo* (Nogueira C and Chin L, unpublished). Consistent with such metastatic phenotype *in vivo*, derivative iMet melanoma cells showed robust invasion activity in response to HGF in Boyden chamber invasion assay *in vitro* (Supplemental Figure S4). Encouraged by this proof of concept validation of IGC, we next utilized this *in vitro* Boyden chamber invasion assay as a first step to examine the additional metastasis candidates in Table 2.

**Figure 3:**
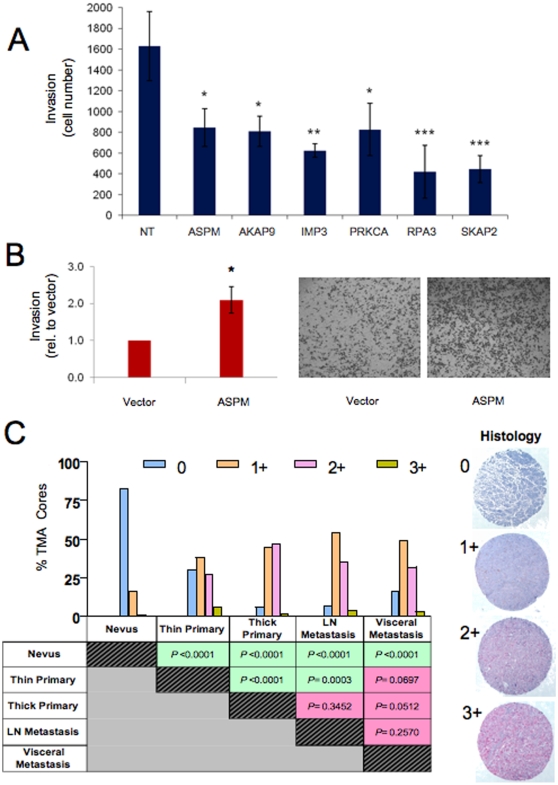
Functional and histopathologic characterization of high-confidence metastasis candidate genes. (**A**) Knockdown of 6 candidate metastasis genes by siRNA inhibited 1205LU Boyden Chamber cell migration. Data represents the average of three replicates. Statistical significance was assessed using a Tukey-Kramer Multiple Comparisons Test, in which each target was compared to the effect of a non-targeting siRNA pool. * = p<0.05; ** = p<0.01; *** = p<0.001. The level of target mRNA knockdown is shown in Supplemental Figure S5. (**B**) Exogenous expression of ASPM enhanced invasion through Matrigel compared to empty vector control on a modified Boyden Chamber assay. Representative images of Boyden chamber assays are shown on the right. Data represent three independent experiments. (**C**) Immunohistochemical survey of Survivin on a melanoma progression tissue microarray. Survivin expression was scored as 0–3+ (see Methods). Percent of TMA cores scored 0 to 3+ for major histopathlogical categories (benign nevi, thin and thick primary cutaneous melanomas, lymph node and visceral metastases) are plotted with p values calculated by χ^2^ test shown in the table below. Representative cores are shown to demonstrate, from top to bottom, intensity of cytoplasmic Survivin expression scored as 0 for no staining, 1+ for mild stain intensity, 2+ for moderate stain intensity, and 3+ for intense stain intensity.

To this end, we selected 6 genes from the candidate list (*ASPM*, *AKAP9*, *IMP3*, *PRKCA*, *RPA3*, and *SKAP2*) based on literature support (see Discussion) to determine whether their knockdown would impact on the invasion of a human melanoma cell, 1205LU. As shown in Figure 3A, siRNA-mediated knockdown of these candidates resulted in a statistically significant inhibition of invasion in the Boyden Chamber assay compared to a non-targeting siRNA oligo pool (p<0.05, p<0.05, p<0.01, p<0.05, p<0.001, p<0.001, respectively). Correspondingly, we also demonstrated that overexpression of *ASPM* in WM3211, a weakly invasive human melanoma cell line, consistently increased invasion through matrix in the Boyden Chamber assay (p<0.05; Figure 3B). Similar results were obtained in a second melanoma cell line, WM115 (data not shown).

### Survivin expression is correlated with progression in human melanoma

It is expected that the putative metastasis genes identified by IGC would exhibit a progression correlated expression pattern in tumor tissues. We utilized a validated commercial antibody against Survivin, an anti-apoptotic protein encoded by *BIRC5*, to perform immunostaining on a melanoma progression tissue microarray (TMA). This TMA contained 480 cores of tumor tissues representing benign nevi, thin and thick primary cutaneous melanoma, as well as lymph node and visceral melanoma metastasis. As shown in Figure 3C, Survivin expression was low to absent in the majority of the benign nevi but was significantly elevated in all melanomas (p<0.0001, χ^2^). Importantly, we observed a significant difference in Survivin expression between cutaneous and metastastic melanomas when comparing thin (but not thick) primary melanomas and lymph node metastases (p = 0.0003, χ^2^). Accordingly, a significant difference in Survivin expression levels was detected between thin and thick primary cutaneous melanomas (p<0.0001, χ^2^), whereas thick primary tumors and lymph node metastases did not show statistically significant differential expression. This pattern of Survivin expression was consistent with the well-known clinical correlation of lymph node spread with thickness of the primary cutaneous lesions, strongly supporting the thesis that the majority of these thick primary melanomas are likely to already have lymph node spread.

## Discussion

Heterogeneity of primary cutaneous melanoma is well appreciated on a number of fronts. Transcriptome profiles have subclassified melanomas by unsupervised methodologies [27], [28], [29], [30], [31]. Somatic mutation frequencies of *BRAF* and *NRAS*, two signature oncogenes in melanoma, exhibit differential preferences for primary tumors arising from different anatomic sites associated with varying UV exposure histories [14]. Through the application of a classification algorithm, we now provide the genome-wide evidence that distinct patterns of copy number aberrations exist in metastatic melanomas. Moreover, these genomic features may potentially stratify patients into cohorts with different clinical outcome, which is not surprising given that melanoma metastasis have also been classified transcriptionally into poor and good outcome subgroups [18]. While we recognize that our sample set was not sufficiently large to draw conclusion on the prognostic significance of these genomic subclasses, the provocative data does suggest that genomics-based prognostic biomarkers can be defined and, therefore, should encourage comprehensive genome characterization of large clinically annotated patient cohorts as a first step toward identification of such DNA-based biomarker(s) for patient stratification.

The importance of recognizing and accounting for tumor heterogeneity in molecular studies is highlighted by the observation that a progression correlated pattern of Survivin expression was only evident when thin and thick cutaneous melanomas were stratified in the analyses of Survivin TMA-IHC data. Along this line, it is intriguing that the Survivin expression difference between thin primary and lymph node metastases was not preserved between thin primary and visceral metastases (χ^2^ p = 0.0697, Figure 3C). This is unexpected if one assumes that visceral metastases progress from lymph node metastases, as suggested by the traditional linear model of melanoma progression. Instead, this data raises the possibility that metastatic spread to lymph nodes and to visceral organs might be driven by distinct molecular pathways. Interestingly, Survivin and HGF/MET, both represented in our IGC-derived metastasis list, were found to cooperate in promoting lymph node and lung metastases in a mouse transgenic model [32]. Our observation that the expression of metastases genes, such as Survivin, appears to be significantly altered when comparing thin and thick primary cutaneous melanomas also highlights the potential need to sub-stratify melanomas based on thickness in future IGC analyses, as these might represent two genetically- and clinically-distinct disease subtypes.

The integrative approach utilized here where two clinical subtypes (primary vs. metastases) were compared on both genome-wide copy number and expression levels is a productive methodology for identifying metastasis-relevant genes, as reflected by our ability to define a short list of candidates that included *MET* receptor tyrosinase kinase and *BIRC5*. The veracity of IGC was further supported by validation of 6 additional candidates selected from the list based on their cancer-relevant roles in other tumor types. U3 small nucleolar ribonucleoprotein (IMP3) and protein kinase C alpha (PRKCA) had been previously linked to aggressiveness and metastasis in a variety of tumor types, including breast, colon, renal cell, lung, ovarian, and hepatocellular cancer [33], [34], [35], [36], [37], [38]. Similarly, A-kinase anchor protein 9 (AKAP9), replication protein A3 (RPA3) and SRC kinase associated phosphoprotein 2 (SKAP2) were enlisted into invasion assay since, although they had been linked to breast, lung, head and neck and pancreatic cancer [39], [40], [41], these genes have not been previously associated with tumor invasion. By virtue of its unbiased nature, IGC also identified unexpected candidates, such as tissue factor pathway inhibitor 2 (TFPI2) and secreted frizzled-related protein 4 (SFRP4). TFPI2 is a serine protease inhibitor in the extracellular matrix that is known to be heavily methylated in an assortment of cancers, including melanoma [42]. While its expression was low in majority of the samples, TFPI2 was gained and overexpressed in 3 out of 72 metastases in our dataset. Similarly, SFRP4, a member of the secreted frizzled-related protein family and a negative regulator of the Wnt pathway that is frequently epigenetically silenced in various tumor types [43], [44] was observed to be gained and overexpressed in 4 of 72 of metastastic melanomas in this study. These patterns suggest unique subgroups of melanomas in which these two genes might serve pro-metastasis roles that are presently unrecognized, much like the example of MITF, a lineage transcription factor that is commonly downregulated during melanoma progression except in a specific subset where MITF is amplified [45].

Although ASPM was part of a signature of 254 genes predictive of metastasis [46], a functional role for this gene in metastatic progression is not obvious given its known role as a spindle protein that regulates brain size with mutations in the gene being associated with microcephaly [47]. The report of ASPM knockdown inhibiting glioblastoma cell growth and neural stem cell self-renewal [48] point to proliferative and survival roles for this gene. Here we uncovered a pro-invasive role for ASPM in melanoma cells. In this regard, it is worth noting that ASPM maps to 1q32, a region that is commonly gained in various solid tumors, including melanoma [13] and metastatic squamous cell carcinomas of the lung [49]. Importantly, 1q gain has been associated with aggressive disease and metastasis in renal clear cell carcinomas [50], hepatocellular carcinoma [51] and papillary thyroid carcinoma [52]. In primary gastric adenocarcinoma, 1q32 status has been significantly correlated with lymph node status [53], and 1q32 gain has been reported to be a prognostic marker in a subset of treatment refractory breast cancers [54]. In summary, these genomic data and preliminary functional characterization on a short list of metastasis candidates encourage their enlistment into in-depth functional, clinicopathological and mechanistic studies.

## Materials and Methods

### Ethics Statement

All research involving human participants was approved by the institutional review boards and granted an exemption. Informed written patient consent was obtained for all tissues used in this study.

### Melanoma samples and DNA extraction

The primary and metastatic melanoma samples analyzed in this study were obtained from three centers: The Medical University of Vienna, Austria (Supplemental Table S1), the Memorial Sloan Kettering Cancer Center of New York, NY and the Brigham and Women's Hospital, Boston, MA (Supplemental Table S2). Complete sample and clinical annotation can be found in Supplemental Table S1 and S2. Frozen tissue sections were prepared and manually macrodissected to obtain an enrichment of greater than 80% tumor cellularity. Genomic DNA from tissue and cell lines was extracted using DNeasy Tissue Kit (Qiagen, Valencia CA). All tumor sample DNA from the Vienna series were subjected to whole genome amplification (WGA) using the REPLI-g Kit (Qiagen) to obtain enough material for aCGH hybridization, while none of the Memorial Sloan Kettering Cancer Center samples and Brigham and Women's Hospital samples was subjected to WGA.

### Array CGH profiling on oligonucleotide microarrays

Genomic DNA was fragmented and random-prime labeled as described previously [11] and hybridized to oligonucleotide arrays containing 22,500 elements designed for expression profiling (Human 1A V2, Agilent Technologies). All data is MIAME compliant, and the raw data has been deposited in to GEO under super-series accession #GSE7606. Using NCBI Build 35, 16,097 unique map positions were defined with a median interval between mapped elements of 54.8 Kb. Fluorescence ratios of scanned images were calculated as the average of two paired arrays (dye swap), and the raw profiles were processed to identify statistically significant transitions in copy number using Circular Binary Segmentation [11], [12]. Each segment was assigned a value that is the median of the log2 ratios of the spanned probes. The data were centered by the tallest mode in the distribution of the segment values. After mode-centering, we defined gains and losses as log2 ratios ≥0.15 or ≤−0.15 (±6 SD of the middle 75% quantile of data) and amplification and deletion as a ratio ≥0.4 or ≤−0.4 (representing 4 and 94% quantiles), respectively.

High-priority MCRs (see [11]) were chosen by requiring at least two samples to show a CNA event and at least one sample to show an extreme CNA event, defined by thresholds +1 and −1, and size of the MCRs was less than 2 MB. The MCRs were mapped to known regions of germline copy number variation (CNV), and CNV status was noted in Tables 1 and 2. Since well-validated cancer relevant genes have been known to harbor germline CNVs [25] we did not exclude candidates that are resident within these regions of known CNV.

### gNMF and Fisher's Exact Test

Genomic NMF was applied to the current dataset as previously described [16]. Briefly, the segmented dataset was first dimension-reduced by eliminating redundant probe locations and then transformed to non-negative values. The resultant dataset was a non-negative matrix, which was subject to gNMF using a custom software package [17] and run in MATLAB (The MathWorks, Inc., Natick, MA). For each factor level two through six, gNMF was repeated 100 times to build a consensus matrix, and this was used to assign samples to clusters based on the most common consensus. The rank K = 3 clustering was further tested for significance by permuting sample labels for secondary samples independently for each chromosome. One hundred permutations were subjected to Rank 3 NMF and the consensus matrix was assessed by cophenetic correlation.

Fisher's Exact Test was used to identify significantly different regional gains or losses between primary and metastastic melanoma. For each aCGH R-segment, each sample was classified as being copy number normal, gained or lost based on log_2_ ratio thresholds of +/−0.15. Two-by-two contingency tables tested gained vs. normal and lost vs. normal between primary and secondary melanoma. Fisher's Exact Test p-values were corrected for multiple testing (q-value FDR 10%, “qvalue” package for R, http://cran.r-project.org).

### Survivin immunohistochemistry and tissue microarrays

The melanocytic tumor progression TMA was as described previously [5]. TMA blocks were sectioned at ∼4µm and antigen was unmasked in retrieval buffer (0.01M citrate buffer, pH6.0) using a pressure cooker at 125°C. Tissue sections were incubated with a 1/500 dilution of primary anti-Survivin polyclonoal antibody NB500-201 (Novus Biologicals, Littleton, CO) for 2 hours at room temperature followed by StreptAvidin- Biotin labeling. Signal was visualized using Alkaline Phosphatase with Permanent Red substrate (DAKO, Carpinteria, CA). L.M.D. and R.M.N. scored each core by visual microscopic inspection as follows: 0+ for no staining and no background; 1+ for weak blush of cytoplasmic staining; 2+ for moderately intense granular cytoplasmic staining; 3+ for markedly intense granular cytoplasmic staining. Most of the cores showed expression in more than 75% of the tumor cells; therefore the staining was graded on intensity rather than % of positive tumor. Statistical comparisons of Survivin IHC staining were performed using a Chi Square test corrected for multiple testing.

### Invasion assays in Boyden Chamber

For exogenous expression of ASPM in WM3211 and WM115 cells, a Gateway® (Invitrogen, Carlsbad, CA) entry clone containing the ASPM cDNA variant BC034607 was obtained from the Center for Cancer Systems Biology (DFCI) and was recombined into pLenti6 V5/DEST (Invitrogen) for virus production and cell transduction following the manufacture's suggestions. For RNAi experiments, 1205LU cells were transfected with Dharmacon SMART siRNA oligo pools (Thermo Fisher Scientific, Lafayette, CO) designed against ASPM, AKAP9, IMP3, PRKCA, RPA3, or SKAP2, as described previously [55]. Boyden Chamber assays were utilized to assess the invasiveness of tumor cells, as one measure of metastatic propensity, following the manufacture's suggestions (BD biosciences, San Jose, CA). Briefly, WM3211, WM115, or 1205LU cells were trypsinized, rinsed twice with PBS, resuspended in serum-free RPMI 1640 medium. Cells were then seeded at a density of 2.5×10^4^ cells/ well in triplicate in 96-well chamber format for ASPM overexpression studies, or at 1.5×10^5^ cells/well in triplicate in 24-well chamber format for siRNA experiments, and the cells were placed in the 10% serum-containing media that served as a chemo-attractant. In parallel, the same number of cells was plated in a same area in regular cell culture plates and grown for the same length of time to serve as input control. Following 20 hrs (ASPM overexpression) or 16 hrs (siRNA experiments) of incubation, cells that had migrated through the chamber were fixed in 10% formalin in PBS, stained with crystal violet and photographed, and cell numbers were counted using an Adobe Photoshop (Adobe Systems, Inc., San Jose, CA) add-on computer program. For analyses of Met induced invasion, boyden chambers were seeded with 5×10^4^ iMet tumor cells in serum-free media. Chambers were placed in chemo-attractant (media containing 10% serum) without and with 50 ng/ml recombinant HGF and incubated for 24 hrs. Invasive cells were visualized by staining with crystal violet.

## Supporting Information

Figure S1 The primary melanoma genome is less altered relative to the metastatic melanoma genome. Based on the number of breakpoints of each sample's segments exceeding +/−0.15 log2 ratio threshold, the genome instability difference between two groups was calculated using a t test.
(1.17 MB TIF)

Figure S2 KM event-free survival curve for 25 melanoma metastasis patients from all three subgroups; K1 and K2 groups show significantly worse event-free survival than K3 (p = 0.0034). Age and sex are not correlated with the three subgroups, which was indicated by non-enrichment using Fisher's Exact Test (data not shown). The numbers of male patients and female patients were tested for enrichment in all three subgroups using Fisher's Exact Test; similarly, patients were divided into young and old groups by median age and tested for enrichment in all three subgroups.
(1.17 MB TIF)

Figure S3 Metastatic Melanoma MCRs were enriched for G1/S genes. Genes mapping within metastatic melanoma MCR boundaries were analyzed in GeneGo software (St Joseph, MI) and a significant number was represented in the MetaCore™ G1/S network (p<0.01). The genes included p14ARF, p16INK4A and p15INK4B, all of which were deleted in metastases (blue circles), and CDK4 and MDM2 were both amplified in metastatic melanoma (red circles). ARHI and GAD45 alpha also mapped to regions of gain/amplification in metastatic melanoma (red circles). A green line denotes activation and red and blue lines signify inhibition of activity. For example, p14ARF inhibits MDM2, which, in turn, activates Ubiquitin and inhibits GADD45 alpha.
(1.17 MB TIF)

Figure S4 Met activation promotes cell invasion. Boyden chambers were seeded with 5×104 iMet tumor cells in serum-free media. Chambers were placed in chemo-attractant (media containing 10% serum) without and with 50 ng/ml recombinant HGF and incubated for 24 hrs. Invasive cells were visualized by staining with crystal violet.
(1.17 MB TIF)

Figure S5 Quantitative PCR assessment of levels of mRNA knockdown of ASPM, AKAP9, IMP3, PRKCA, RPA3 and SKAP2 in 1205LU cells following transfection of siRNA oligo pools. % mRNA knockdown is relative to transcript levels after transfection of a non-targeting siRNA pool (see methods). Ranges in knockdown levels reflect standard deviations from three replicates.
(1.17 MB TIF)

Table S1 Sample annotation and clinical Information on melanoma samples from Medical University of Vienna, Austria.
(0.04 MB DOC)

Table S2 Annotation of samples from Memorial Sloan Kettering and the Brigham and Women's Hospital.
(0.03 MB DOC)
