# Supplementary figures and images for: Integrative Genome Comparison of Primary and Metastatic Melanomas
Source: PLoS One. 2010 May 24;5(5):e10770. doi: 10.1371/journal.pone.0010770 (PMC2875381; doi:10.1371/journal.pone.0010770)

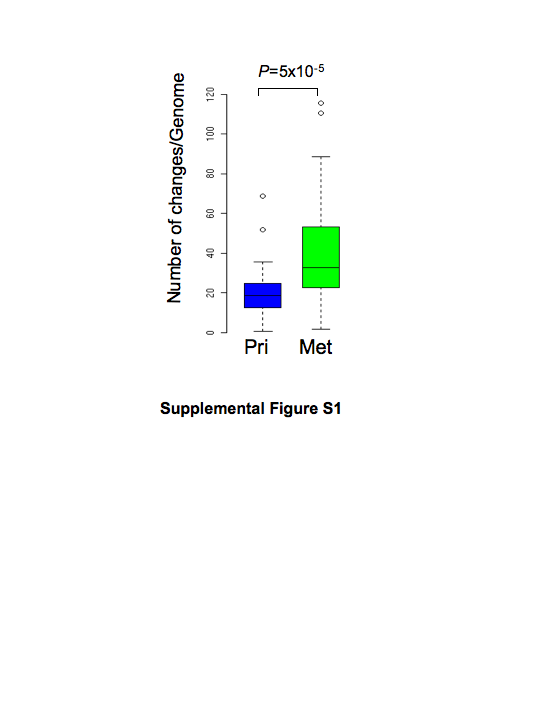

Supplement: Figure S1 — The primary melanoma genome is less altered relative to the metastatic melanoma genome. Based on the number of breakpoints of each sample's segments exceeding +/−0.15 log2 ratio threshold, the genome instability difference between two groups was calculated using a t test. (1.17 MB TIF) [file pone.0010770.s001.tif]

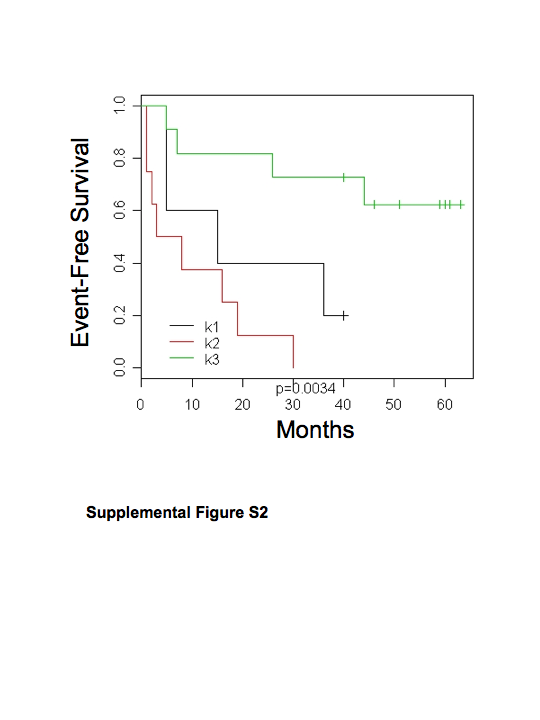

Supplement: Figure S2 — KM event-free survival curve for 25 melanoma metastasis patients from all three subgroups; K1 and K2 groups show significantly worse event-free survival than K3 (p = 0.0034). Age and sex are not correlated with the three subgroups, which was indicated by non-enrichment using Fisher's Exact Test (data not shown). The numbers of male patients and female patients were tested for enrichment in all three subgroups using Fisher's Exact Test; similarly, patients were divided into young and old groups by median age and tested for enrichment in all three subgroups. (1.17 MB TIF) [file pone.0010770.s002.tif]

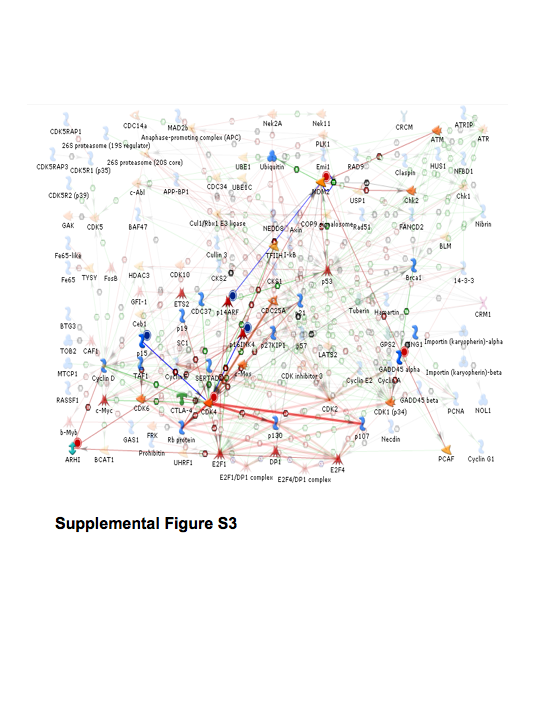

Supplement: Figure S3 — Metastatic Melanoma MCRs were enriched for G1/S genes. Genes mapping within metastatic melanoma MCR boundaries were analyzed in GeneGo software (St Joseph, MI) and a significant number was represented in the MetaCore™ G1/S network (p<0.01). The genes included p14ARF, p16INK4A and p15INK4B, all of which were deleted in metastases (blue circles), and CDK4 and MDM2 were both amplified in metastatic melanoma (red circles). ARHI and GAD45 alpha also mapped to regions of gain/amplification in metastatic melanoma (red circles). A green line denotes activation and red and blue lines signify inhibition of activity. For example, p14ARF inhibits MDM2, which, in turn, activates Ubiquitin and inhibits GADD45 alpha. (1.17 MB TIF) [file pone.0010770.s003.tif]

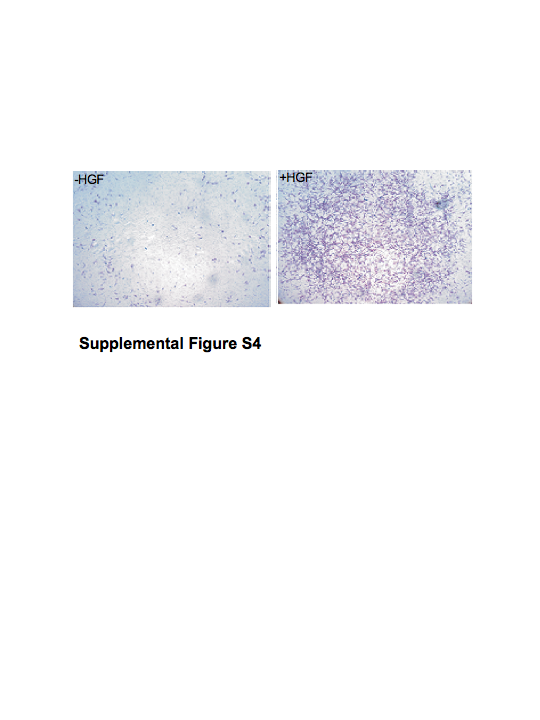

Supplement: Figure S4 — Met activation promotes cell invasion. Boyden chambers were seeded with 5×10^4^ iMet tumor cells in serum-free media. Chambers were placed in chemo-attractant (media containing 10% serum) without and with 50 ng/ml recombinant HGF and incubated for 24 hrs. Invasive cells were visualized by staining with crystal violet. (1.17 MB TIF) [file pone.0010770.s004.tif]

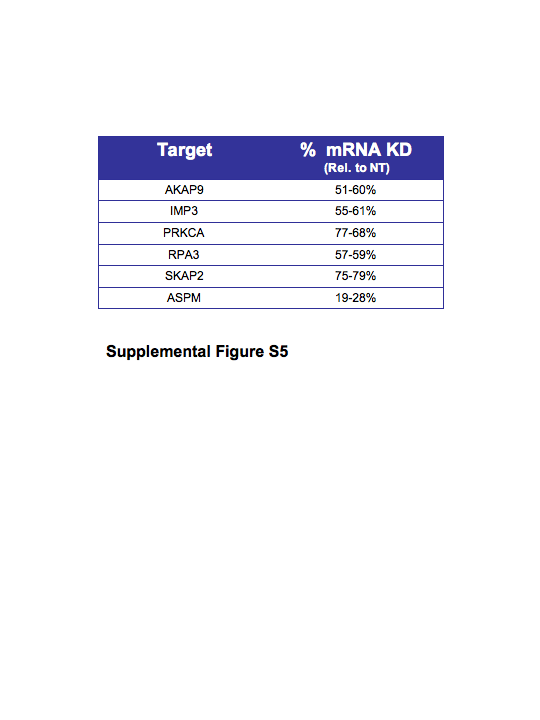

Supplement: Figure S5 — Quantitative PCR assessment of levels of mRNA knockdown of ASPM, AKAP9, IMP3, PRKCA, RPA3 and SKAP2 in 1205LU cells following transfection of siRNA oligo pools. % mRNA knockdown is relative to transcript levels after transfection of a non-targeting siRNA pool (see methods). Ranges in knockdown levels reflect standard deviations from three replicates. (1.17 MB TIF) [file pone.0010770.s005.tif]
