# Supplementary material for: Integrative Genome Comparison of Primary and Metastatic Melanomas
Source: PLoS One. 2010 May 24;5(5):e10770. doi: 10.1371/journal.pone.0010770 (PMC2875381; doi:10.1371/journal.pone.0010770)
Supplement: Table S1 — Sample annotation and clinical Information on melanoma samples from Medical University of Vienna, Austria. (0.04 MB DOC) [file pone.0010770.s006.doc]

Supplemental Table S1. Sample annotation and clinical Information on melanoma samples from Medical University of Vienna, Austria.
