# Supplementary material for: Integrative Genome Comparison of Primary and Metastatic Melanomas
Source: PLoS One. 2010 May 24;5(5):e10770. doi: 10.1371/journal.pone.0010770 (PMC2875381; doi:10.1371/journal.pone.0010770)
Supplement: Table S2 — Annotation of samples from Memorial Sloan Kettering and the Brigham and Women's Hospital. (0.03 MB DOC) [file pone.0010770.s007.doc]

Supplemental Table S2. Annotation of samples from Memorial Sloan Kettering and the Brigham and Women’s Hospital.
